# Materials for Fillings

**Published:** 1878-12

**Authors:** 


### MATERIALS FOR FILLINGS.

BY DE-CE-AICH.

My patients often ask me what I fill teeth with. I generally answer, "Nearly everything." I should no more think of filling all teeth with one and the same material than I should expect to restore every edentulous mouth with a single pattern of White's gum teeth. I decide what material to use for my filling after examining the cavity, not before, and I permit the conditions in and about the cavity, that is the environment, to decide for me what kind of filling I want. Perhaps the qualities that would be most desirable in a filling for the cavity under consideration, are not found combined in any available material. In this case I make use of that kind of filling which will most nearly fulfill the indications. Sometimes I find myself in accord with the "new departure." Sometimes I form my fillings on classical models, from the shining sands of California. Gutta percha, cement plombe, or oxy-chloride of zinc, the amalgams, from Townsend's to Fletcher's, and even sandarac, wax, plaster of Paris and resin, are each and all brought into requisition. I have never yet been guilty of the consummate folly of supposing that plastic fillings are invariably better than gold or tin. And I expect to die before I see the day in which I can regard gold and tin as invariably better than the various cements and amalgams for every case. Curiously enough, however, I have generally found that I can preserve cavities of decay pretty well with gold, especially if I can persuade the patient to practice pretty thorough cleansing. But then, my main practice has been conducted in a single small town in America, and but for a very limited period. Perhaps if I had lived in St. Louis or Philadelphia I should have found it possible to save teeth, according to the principles of the new departure."

